# Effects of prenatal maternal smoking on placental IGF-1, leptin, and HPL expression and breastfeeding practices

**DOI:** 10.1186/s12884-026-08726-w

**Published:** 2026-02-07

**Authors:** Hayrunnisa Yeşil Sarsmaz, Seren Gülşen Gürgen, Kemal Sarsmaz, Oya Sayın

**Affiliations:** 1 Department of Histology and Embryology, Faculty of Health Sciences, Manisa Celal Bayar University, Uncubozkoy, Manisa, Turkey; 2 Department of Histology and Embryology, Vocational School of Health Services, Manisa Celal Bayar University, Manisa, Turkey; 3 Department of Obstetrics and Gynecology, Faculty of Medicine, Manisa Celal Bayar University, Manisa, Turkey; 4 Department of Biochemistry, Vocational School of Health Service, Dokuz Eylul University, Izmir, Turkey

**Keywords:** Smoking, Placenta, IGF-1, Leptin, HPL, Breastfeeding practices

## Abstract

Smoking during pregnancy has many negative effects, such as growth retardation, premature birth, and placental detachment. However, although it is known to have negative effects during breastfeeding, it has not been examined in detail as the pregnancy period. This study aimed to investigate the effects of smoking during pregnancy on the placenta and its relationship with breastfeeding practices and placental hormone levels. The study included 70 women who underwent spontaneous vaginal delivery. The participants were divided into two groups: smokers (*n* = 35) and non-smokers (*n* = 35). The breastfeeding conditions of both groups were evaluated prior to discharge and on the 10th day, postpartum. Cross-sectional samples of placental tissues were collected, and their IGF-1, leptin, and HPL immunoreactivities were analyzed. A significant difference was found between the smoking status of the mothers and the immunoreactivity of IGF-1, leptin, and HPL (*p* < 0.05). Pregnant mothers who smoked showed lower expression of IGF-1, leptin, and HPL than non-smoker pregnant mothers. These results indicate that maternal smoking during pregnancy has adverse effects on the placenta and cord blood and may affect fetal development. Moreover, prenatal maternal smoking is associated with changes in breastfeeding practices.

## Introduction

Maternal smoking remains a major public health problem worldwide and continues to affect a substantial proportion of women of reproductive age [1–4]. Although global smoking prevalence has declined over recent decades, tobacco use among pregnant women persists, particularly in certain regions, and is associated with adverse perinatal outcomes [5–9]. One of the most consistently reported fetal consequences of prenatal smoking exposure is impaired fetal growth, including intrauterine growth restriction and an increased risk of small for gestational-age birth, largely mediated by chronic hypoxia, carbon monoxide exposure, and placental dysfunction [9, 10]. In addition, maternal smoking has been linked to reduced exclusive breastfeeding rates and delayed initiation of lactation, potentially through both behavioral and biological mechanisms [11–19]. Importantly, accumulating evidence suggests that some of the biological effects of smoking on both fetal growth and early postnatal feeding may be mediated through alterations in placental endocrine function. However, the biological pathways connecting prenatal smoking, placental hormonal regulation, and early postnatal feeding outcomes remain incompletely understood.

The placenta plays a central role in regulating fetal growth and maternal metabolic adaptation through the synthesis of key hormones, including insulin-like growth factor-1 (IGF-1), leptin, and human placental lactogen (HPL) [20–26]. IGF-1 is a critical mediator of cellular proliferation, placental blood flow, and fetal tissue development [20, 21]. Placental leptin contributes to energy homeostasis and trophoblast function and may reflect adaptive responses to placental stress in growth restricted pregnancies [23, 24]. HPL, a growth hormone and prolactin-like peptide, increases progressively during gestation in proportion to placental mass and supports fetal growth by modulating maternal glucose and lipid metabolism [26]. Beyond their roles in fetal growth, these hormones are also involved in maternal metabolic and neuroendocrine adaptations that are essential for the initiation and maintenance of lactation. Although maternal smoking is known to impair placental vascularization and endocrine activity, its specific effects on placental IGF-1, leptin, and HPL expression and their potential downstream implications for both fetal development and lactogenesis have not been fully elucidated.

In parallel with its effects on fetal growth, maternal smoking has been consistently associated with suboptimal breastfeeding practices, including delayed initiation and reduced exclusivity, potentially mediated by neuroendocrine and metabolic alterations affecting lactogenesis and milk composition [10–19]. However, whether smoking-related alterations in placental endocrine function particularly in hormones involved in both fetal growth and lactogenesis coexist with differences in early breastfeeding behavior has not been systematically investigated.

Therefore, the aim of the present study was to evaluate the association between maternal smoking during pregnancy and placental as well as umbilical cord blood levels of IGF-1, leptin, and HPL, and to examine whether maternal smoking is related to differences in early breastfeeding practices during the immediate postpartum period.

## Materials and methods

### Ethical approval

This study was approved by the Manisa Celal Bayar University Health Sciences Ethics Committee (Approval date: 02 August 2017; Decision No: 20.478.486).

### Study design and participants

This prospective case–control study included 70 postpartum women (35 smokers and 35 non-smokers) who delivered via spontaneous vaginal birth at Merkezefendi State Hospital between October 2017 and April 2018 [27]. Written informed consent was obtained from all participants before enrollment. Participants completed a questionnaire related to demographics and breastfeeding.

The participants were categorized into two groups:

- Group 1 (smokers): Women who smoked at least 100 cigarettes in their lifetime and continued smoking before and during pregnancy (*n* = 35).
- Group 2 (non-smokers): Women who had never smoked in their lifetime and did not smoke during pregnancy (*n* = 35).

Breastfeeding status was assessed prior to hospital discharge and on postpartum day 10.

### Inclusion and exclusion criteria for research

Pregnant mothers who had smoked at least 100 cigarettes during their lifetime, smoked during pregnancy, gave birth spontaneously, had no systemic diseases, and did not have any health condition that could prevent breastfeeding were included in the study group. The study also included the use of e-cigarettes and vapes by the participants.

Those who had never smoked, had spontaneous vaginal birth, had no systemic disease (diabetes mellitus, hypertension, rheumatoid arthritis, systemic lupus erythematosus, multiple sclerosis, autoimmune diseases in general, systemic infections such as sepsis, and chronic cardiovascular conditions such as heart failure), and did not have any health condition that could interfere with breastfeeding were included in the control group.

Pregnant mothers with multiple pregnancies, preeclampsia, pregestational or gestational diabetes, thyroid dysfunction, preterm premature rupture of membranes (PPROM), or foreign nationality were excluded from the study.

### Placental tissue collection and processing

Immediately after delivery, 35 placental tissue samples per group were collected. The placentas were rinsed with phosphate-buffered saline (PBS) to remove excess blood. Tissue samples (~ 1 cm³) were collected from standardized locations on both the maternal and fetal surfaces near the peripheral margin. Samples were fixed in 10% neutral-buffered formalin for 24–48 h, dehydrated through graded ethanol, and embedded in paraffin blocks. Sections of 5 μm thickness were cut using a microtome (Leica RM 2135, Germany) for immunohistochemical analyses.

### Immunohistochemical staining on placenta

A 1 cm³ tissue sample was obtained from both the fetal and maternal surfaces of the placenta, specifically from the peripheral margin, and was then placed in 10% formaldehyde and fixed for 24 h. Samples were collected from the same region of the placenta. The samples were then subjected to dehydration and embedded in paraffin blocks. The embedded tissues were transferred as 5 μm sections onto a microscope slide using a microtome (Leica RM 2135; Bensheim, Germany). For immunohistochemical staining, the sections were first incubated at 60 °C overnight and then incubated in xylene for 30 min. After washing with serial concentrations of ethanol (95, 80, 70, and 60%), the sections were washed with distilled water and phosphate-buffered saline (PBS) for 10 min. They were then incubated in 2% trypsin in Tris buffer at 37 °C for 15 min and washed with PBS three times for 5 min. The sections were incubated in a 3% H_2_O_2_ solution for 15 min to inhibit endogenous peroxidase activity. The sections were then washed with PBS and incubated with the blocking solution, Large Volume Ultra V Block (Thermo Fisher, USA) for 1 h. The sections were then incubated at 4 °C with primary antibodies overnight: HPL (1:100, mouse monoclonal, Thermo Fisher, USA), leptin (1:100, Bioss, Woburn, Massachusetts, USA), IGF-1 (1:100, Bioss, Woburn, Massachusetts, USA). The cells were then washed three times for 5 min each with PBS, followed by incubation with biotinylated IgG and streptavidin–peroxidase conjugate (Thermo Fisher, CA, USA). After washing three times for 5 min with PBS, the sections were incubated with diaminobenzidine (DAB) substrate (Thermo Fisher, CA, USA) for 5 min to detect immunoreactivity, followed by Mayer’s hematoxylin. The sections were covered with a mounting medium and analyzed under a light microscope (CX 31; Olympus, Tokyo, Japan). Negative control samples were processed in an identical manner, but without the primary antibody step. All of the immunostained sections were reviewed by two histologists, who had no knowledge of the groups.The slides were first examined under low magnification (x10 objective) to identify regions with HPL, leptin and IGF-1 staining. Ten random areas were selected and scored for each section. In sections where staining was particularly intense, a single random field was chosen for detailed analysis. The proportion of placenta in each selected field was determined by counting at high magnification. At least 100 cells were scored per field at × 40 magnification for each group. All sections were scored semi-quantitatively, considering both the intensity and percentage of cells stained at each intensity. The intensities were classified as 0 (no staining), 1 (weak staining), 2 (moderate staining), and 3 (strong staining), whereas 10% groupings were used for the percentage of positively stained cells. For each slide, a value designated as H-SCORE was obtained by applying the following algorithm: H-SCORE = Σ Pi.(i + 1), where i and Pi represent the intensity and percentage of cells stained at each intensity. The corresponding H-SCOREs were calculated separately. Numbers were calculated on 10 randomly selected areas using the image analysis system (Leica Q Win V3 Plus Image), and the mean values were employed for analysis [28].

### Umbilical cord blood collection and biochemical analysis

Whole blood collected from the umbilical cord vein was drawn into 5.5 mL serum separator tubes with yellow caps (Product No: #30011181, Hawach Scientific) for biomarker analysis. The samples were kept at room temperature for at least 30 min to allow coagulation. The tubes were then centrifuged at 1000 x g for 15 min at + 4 °C, and the serum was separated. The obtained serum samples were aliquoted into 0.5 mL polypropylene Eppendorf tubes, one for each biomarker. Samples were stabilized at − 80 °C and stored in a deep freezer until the day of analysis. Once the planned number of patients was reached, the samples were thawed homogeneously by placing them at 4 °C overnight. Prior to the analyses, leptin and human placental lactogen (HPL) levels were measured using the sandwich ELISA method described above. The analysis was performed according to the manufacturer’s protocol using a commercially available ELISA kit (Elabscience; catalog number: E-EL-H6017). Each sample was analyzed in duplicate. Standards and samples were added to 96-well plates and incubated at room temperature for 2 h. The wells were washed with PBS containing 0.05% Tween-20, followed by the addition of a biotin-labeled secondary antibody and incubation for 1 h. After washing, streptavidin-HRP conjugate was added and incubated for 30 min. Color development was achieved by adding 3, 3 ′, 5, 5′-tetramethylbenzidine (TMB) substrate and incubating in the dark for 15 min. The reaction was stopped using 2 N sulfuric acid, and the optical density was measured at 450 nm using a microplate reader (BioTek ELx800). The results were quantified using a standard curve based on a logarithmic regression analysis. Each plate included internal positive and negative control. All samples were analyzed simultaneously under the same conditions to ensure that the half-lives of the biomarkers were unaffected. The coefficient of variation- CV (%) for the replicates was maintained below 10% to ensure measurement reliability [29]. Due to budgetary constraints, the IGF ELISA kit could not be procured; thus, IGF analysis was not performed.

### Statistics

A priori sample size estimation was performed using G*Power version 3.1.9.7 for a two-sided comparison of two independent means with equal group allocation. The primary outcome used for the calculation was the difference in placental hormone expression levels between smoking and non-smoking mothers.

Based on previous lactation- and placenta-related studies reporting large differences in hormone concentrations between smokers and non-smokers, a large effect size was assumed (Cohen’s d = 1.15). This value reflects a conservative representation of large effects described in the literature and was not derived post hoc from the present dataset. Using this assumed effect size with an alpha level of 0.05 and 95% power, the minimum required sample size was approximately 20 participants per group [30, 31]. Therefore, enrolling 35 participants per group provided sufficient statistical power to detect large between-group differences.

All statistical analyses were performed using SPSS version 25.0 (SPSS Inc., Chicago, IL, USA). Continuous variables were expressed as mean ± standard deviation. Group comparisons were conducted using independent samples t-tests or one-way ANOVA, categorical variables were analyzed using chi-square tests, and linear regression analyses were applied where appropriate. Statistical significance was defined as *p* < 0.05.

## Results

### Study population

A total of 70 women (35 smokers and 35 non-smokers) who delivered via spontaneous vaginal birth were included in this study. Demographic and breastfeeding-related characteristics, placental immunohistochemical markers (IGF-1, leptin, and HPL), and umbilical cord blood biochemical levels of leptin and HPL were evaluated for each participant. All collected placental tissue samples (*n* = 70) were adequate for histological and immunohistochemical assessment, and all cord blood samples were suitable for biochemical analyses.

### Demographic characteristics

The sociodemographic characteristics of the participants are presented in Table 1. Birth weight percentiles were classified as ≤ 10th percentile (small for gestational age, SGA) or > 10th percentile (non-SGA). SGA was significantly more common among infants born to mothers who smoked (75%, *n* = 18) than among those born to non-smokers (25%, *n* = 6) (χ² = 9.13, *p* = 0.003). No statistically significant differences were found between the groups with respect to maternal age, marital status, education level, parity, abortion history, number of living children, infant sex, placental expulsion time (min), or placental weight (g) (*p* > 0.05).

**Table 1:** Sociodemographic and obstetric variables of the study population (*n* = 70)

Variable	Smokers (*n* = 35)		Nonsmokers (*n* = 35)		x^2^/*p*
	Frequency (*n*)	%	Frequency (*n*)	%	
Age (years)					
< 26	20	57.1	14	40	2.05/0.15
≥ 26	15	42.9	21	60	
Marital status					
Married	33	94.3	35	100	-/-
Single	2	5.7	0	0	
Level of education					
No formal education	6	17,1	3	8.6	3.77/0.15
Formal education	15	42.9	10	28.6	
Primary school and above	14	40	22	62.9	
National Public Health Insurance Coverage					
Yes	11	31.4	23	65.7	8.23/ 0.004*
No	24	68.6	12	34.3	
Parity					
None	10	28.6	10	28.6	2.88/*0.23*
1	10	28.6	16	45.7	
2 and above	15	42.9	9	25.7	
Abortions					
None	27	77.1	30	85.7	-/-
1	6	17.1	3	8.6	
2 or more	2	5.7	2	5.7	
Living children					
None	11	31.4	11	31.4	1.34/0.51
1	11	31.4	15	42.9	
2 and above	13	37.1	9	25.7	
Infant sex					
Girl	9	25.8	15	42.9	1.585/0.208
Boy	26	74.2	20	57.1	
Placental expulsion time (min)					
≤ 15 min	34	51.5	32	48.5	1.061/0.303
≤30 min	1	25	3	75	
Placental weight (g)					
400–500 g	5	62.5	3	37.5	6.976/0.073
501–600 g	18	66.7	9	33.3	
601–700 g	7	33.3	14	66.7	
≥ 701	5	35.7	9	64.3	
Birth weight: SGA: ≤10thP	18	75	6	25	9.130/ 0.003*
Non-SGA: >10thP	17	37	29	63	

χ²: Chi-square test. Birth weight percentiles were calculated using the Fetal Biometry 3.0 calculator (Perinatology.com). Infants with birth weight ≤ 10th percentile for gestational age were classified as small for gestational age (SGA), and those with birth weight > 10th percentile as non-SGA

*p < 0.05

**Table 2:** Comparison of gestational age at delivery between smoking and non-smoking mothers (*n* = 70)

Independent T- test	Groups	*N*	Mean	Std. Deviation	t	df	*p*
Gestational Age at Delivery	Smokers	35	36.2	2.5	−2.587	68	0.012**
	NonSmokers	35	37.7	3			

Independent T test

(*) Indicates significant differences between groups

^**^p ≤ 0.05

Comparison of gestational age at delivery revealed a statistically significant difference between smoking and non-smoking mothers (Table 2). The mean gestational age was significantly lower in the smoking group compared with the non-smoking group (36.2 ± 2.5 vs. 37.7 ± 3.0 weeks, respectively; t = − 2.587, df = 68, *p* = 0.012).

**Table 3:** Mean ± SD values of term placenta IGF-1, leptin, and HPL immunohistochemistry scores results between birth weight groups (SGA ≤ 10th percentile; Non-SGA > 10th percentile) (*n* = 70)

Independent T- test	Groups	*N*	Mean	Std. Deviation	t	df	*p*
IGF − 1	SGA:≤10thP	24	103.5	72.8	−2.874	68	0.005**
	Non-SGA: >10thP	46	159.3	79.3			
Leptin	SGA:≤10thP	24	283.1	93.8	3.211	68	0.002**
	Non-SGA: >10thP	46	202.9	101.9			
HPL	SGA:≤10thP	24	149.2	72.8	−2.866	68	0.006**
	Non-SGA: >10thP	46	218.6	79.2			

Independent T test

(*) Indicates significant differences between groups

^**^p ≤ 0.05

Birth weight percentiles were calculated using gestational age–adjusted reference standards, and infants were categorized as SGA (≤ 10th percentile) or non-SGA (> 10th percentile).

When infants were classified according to birth weight percentiles, significant differences in placental hormone expression were observed between the SGA (≤ 10th percentile) and non-SGA (> 10th percentile) groups. Placental IGF-1 immunohistochemical scores were significantly lower in the SGA group compared to the non-SGA group (103.5 ± 72.8 vs. 159.3 ± 79.3; t = − 2.874, *p* = 0.005). Similarly, placental leptin expression differed significantly between groups, with higher scores observed in the SGA group than in the non-SGA group (283.1 ± 93.8 vs. 202.9 ± 101.9; t = 3.211, *p* = 0.002). In addition, placental HPL expression was significantly reduced in the SGA group compared to the non-SGA group (149.2 ± 72.8 vs. 218.6 ± 79.2; t = − 2.866, *p* = 0.006) (Table 3).

To further investigate the combined effects of maternal smoking status and fetal growth status on placental hormone expression, participants were stratified into four groups based on smoking status (smoker vs. non-smoker) and birth weight percentile: smoker–SGA (≤ 10th percentile), smoker–non-SGA (> 10th percentile), non-smoker–SGA, and non-smoker–non-SGA. This stratification allowed for the assessment of both independent and combined effects of smoking exposure and fetal growth status on placental IGF-1, leptin, and HPL expression. One-way ANOVA demonstrated significant differences in placental IGF-1, leptin, and HPL immunohistochemical scores among the four study groups (*p* < 0.001 for all markers) (Table 4).

For all three placental hormones, the lowest expression levels were observed in the smoker–SGA group, whereas the highest levels were detected in the non-smoker–non-SGA group.

**Table 4:** Comparison of placental IGF-1, leptin, and HPL immunohistochemical scores among combined smoking status and fetal growth groups (SGA ≤ 10th percentile; non-SGA > 10th percentile) (*n* = 70)

Anova	Groups	*N*	Mean	Std. Deviation	F	*p*
IGF − 1	Smoker-SGA	18	64.8	24.7	183.07	0.000*
	Smoker-non-SGA	17	63.3	19.1		
	Non-Smoker-SGA	6	219.5	27.7		
	Non-Smoker-non-SGA	29	215.6	31.9		
Leptin	Smoker-SGA	18	330	49.3	153.33	0.000*
	Smoker-non-SGA	17	326.9	47.5		
	Non-Smoker-SGA	6	142.5	24.5		
	Non-Smoker-non-SGA	29	130.1	24.1		
HPL	Smoker-SGA	18	100.6	17.1	188.33	0.000*
	Smoker-non-SGA	17	99.2	22.2		
	Non-Smoker-SGA	6	295	61.5		
	Non-Smoker-non-SGA	29	288.6	38.6		

Anova test

(*) Indicates significant differences between groups

^**^p ≤ 0.05

Post hoc Tukey multiple comparison analysis revealed that both smoker–SGA and smoker–non-SGA groups had significantly lower IGF-1, leptin, and HPL scores compared with their respective non-smoker counterparts (*p* < 0.001 for all comparisons) (Table 5).

**Table 5:** Post hoc multiple comparisons of IGF-1, leptin, and HPL immunohistochemical scores across groups (*n* = 70)

Multiple comparisons between groups *P* Value Posthoc/Tukey	IGF − 1	Leptin	HPL
Smoker-SGA vs. Non-Smoker-SGA	0.000*	0.000*	0.000*
Smoker-SGA vs. Non-Smoker-non-SGA	0.000*	0.000*	0.000*
Smoker-non-SGA vs. Non-Smoker-SGA	0.000*	0.000*	0.000*
Smoker- non-SGA vs. Non-Smoker- non-SGA	0.000*	0.000*	0.000*
Smoker-SGA vs. Smoker- non-SGA	0.998	0.995	0.999
Non-Smoker- SGA vs. Non-Smoker- non-SGA	0.989	0.889	0.974

(*) Indicates significant differences between groups

*p ≤ 0.05

One-way ANOVA revealed statistically significant differences in placental IGF-1, leptin, and human placental lactogen (HPL) immunohistochemical scores among groups stratified by combined maternal smoking status and fetal growth categories (SGA ≤ 10th percentile and non-SGA > 10th percentile) (Table 4). Significant group effects were observed for all three markers (IGF-1: F = 183.07, *p* < 0.001; leptin: F = 153.33, *p* < 0.001; HPL: F = 188.33, *p* < 0.001).

Placental IGF-1 immunoreactivity was markedly reduced in both the smoker–SGA and smoker–non-SGA groups compared with their non-smoker counterparts, whereas substantially higher IGF-1 scores were observed in the non-smoker–SGA and non-smoker–non-SGA groups. These findings indicate a strong suppressive effect of maternal smoking on placental IGF-1 expression, which is largely independent of fetal growth category.

In contrast, leptin immunoreactivity was significantly elevated in the placentas of mothers who smoked in both the SGA and non-SGA groups, while the non-smoker groups exhibited considerably lower leptin scores. This pattern suggests that maternal smoking is associated with the upregulation of placental leptin expression, potentially reflecting a compensatory response to smoking-induced placental stress or hypoxia.

Similarly, HPL immunoreactivity was significantly lower in smokers regardless of fetal growth status, whereas non-smokers, particularly the non-smoker–non-SGA group, demonstrated markedly higher HPL expression. These results imply that maternal smoking adversely affects the placental endocrine capacity and fetal metabolic support mechanisms.

Post hoc multiple comparisons using the Tukey test confirmed that the differences in IGF-1, leptin, and HPL immunohistochemical scores between the smoker and non-smoker groups were statistically significant (*p* < 0.001 for all comparisons) (Table 5). Significant differences were consistently observed between the smoker–SGA and non-smoker–SGA, smoker–SGA and non-smoker–non-SGA, smoker–non-SGA and non-smoker–SGA, and smoker–non-SGA and non-smoker–non-SGA groups. Collectively, these findings demonstrate that maternal smoking status is the predominant determinant of placental IGF-1, leptin, and HPL expression, exceeding the effect of fetal growth classification.

### Linear regression analyses

Linear regression models indicated that maternal smoking had a significant negative effect on birth weight, with infants of smoking mothers weighing, on average, 486 g less, explaining 20.8% of the variance (B = − 0.486, *p* < 0.001). In contrast, maternal age did not have a significant effect on birth weight (*p* = 0.463) and explained only 0.8% of the variance. A positive association was observed between maternal education level and birth weight, suggesting an estimated increase of approximately 437 g with higher education levels; however, this relationship was of borderline statistical significance (B = 0.437, *p* = 0.054). Although social security status showed a positive association with birth weight, this effect did not reach statistical significance (B = 0.163, *p* = 0.151).

A strong and significant association was also identified between gestational age and birth weight, with longer gestational duration being associated with higher birth weight (B = 0.729, *p* = 0.020), accounting for 13.2% of the variance. Collectively, these findings demonstrate that maternal smoking is the most influential determinant of reduced birth weight in this cohort, exceeding the effects of maternal age, education, and socioeconomic indicators, and underscore the critical role of gestational duration in fetal growth and perinatal health outcomes (Table 1).

### Breastfeeding practices

When participants were contacted by phone on the 10th day postpartum to assess characteristics related to breastfeeding practices, it was found that the initiation of breastfeeding within the first hour after birth was lower in smoking mothers (57.1%, *n* = 20) compared to non-smoking mothers (94.3%, *n* = 33). Furthermore, the breastfeeding rate was lower in smoking mothers (62.9%, *n* = 22), while the rate of mothers feeding their babies with both breast milk and formula was higher (37.1%, *n* = 13). Additionally, 91.4% (*n* = 64) had initially given colostrum, 75.7% (*n* = 53) had breastfed within the first hour, 77.1% (*n* = 54) were currently exclusively breastfeeding, and among those who provided supplementary feeding in addition to breast milk, 12.9% (*n* = 9) reported doing so because of infant-related problems (Table 6).

**Table 6:** Breastfeeding practices process -related characteristics/variables of participants (*n* = 70)

Variable	Smokers (*n* = 35)		Nonsmokers (*n* = 35)		Total		x^2^/*p*
	Frequency (*n*)	%	Frequency (*n*)	%	Frequency (*n*)	%	
The first food you give to your child orally							
Colostrum	31	88.6	33	94.3	64	91.4	-/-
Formula milk	4	11.4	2	5.7	6	8.6	
Time to initiate feeding after birth							
Within the first hour	20	57.1	33	94.3	53	75.7	13.130/0.000*
Within the first two hours	15	42.9	2	5.7	17	24.3	
The breastfeeding status of the baby							
Only breast milk	22	62.9	32	91.4	54	77.1	8.102/0.004*
Breast milk and formula	13	37.1	3	8.6	16	22.9	
Reasons for Supplementary Feeding (*n* = 16)							
Insufficient milk supply	3	8.6	0	0	3	4.3	-/-
Baby refuses to breastfeed	3	8.6	0	0	3	4.3	
Maternal health issues	1	2.9	0	0	1	1.4	
Infant health issues	6	17.1	3	8.6	9	12.9	

*p < 0.05 × 2: Chi-square test

### Immunohistochemical findings

**Fig. 1:**
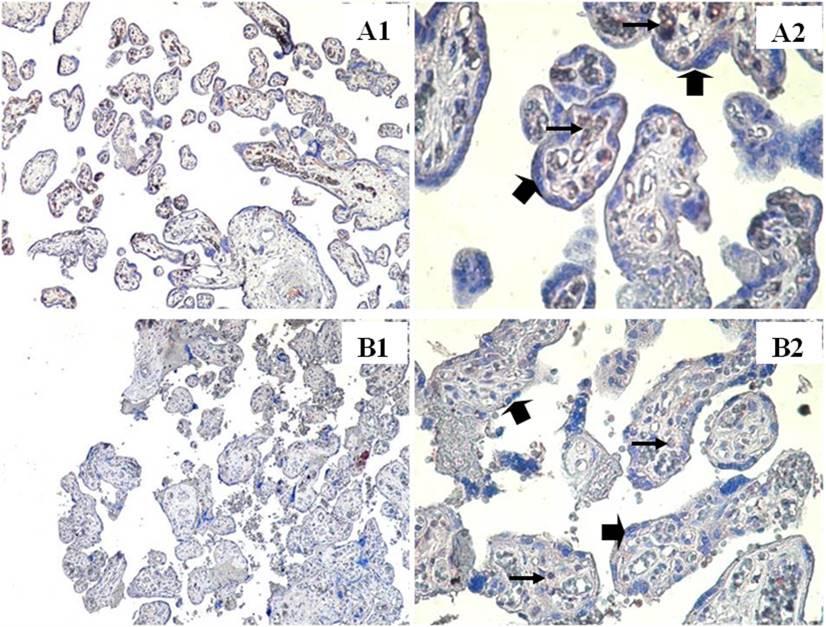
IGF-1 immunostainings in the placenta villi of smoker and nonsmoker groups. Nonsmoker **A**, Smoker **B**. ➠:Villous syncytiotrophoblast cells, →: villous stromal cells. (A1,B1) X100, (A2,B2) X400

**Fig. 2:**
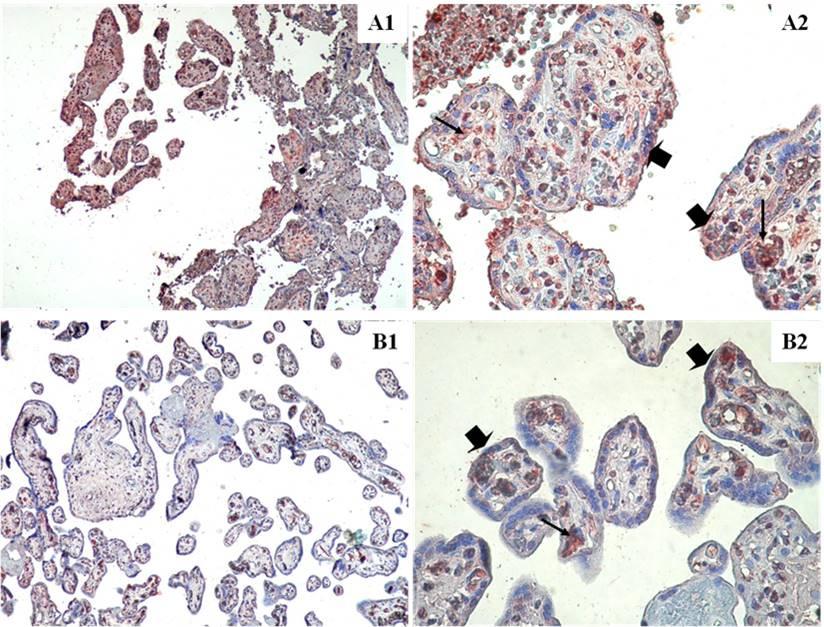
Leptin immunostainings in the term placenta villi of smoker and nonsmoker groups. Nonsmoker **A**, Smoker **B**. ➠:Villous syncytiotrophoblast cells, →: villous stromal cell. (A1,B1) X100, (A2,B2) X400

**Fig. 3:**
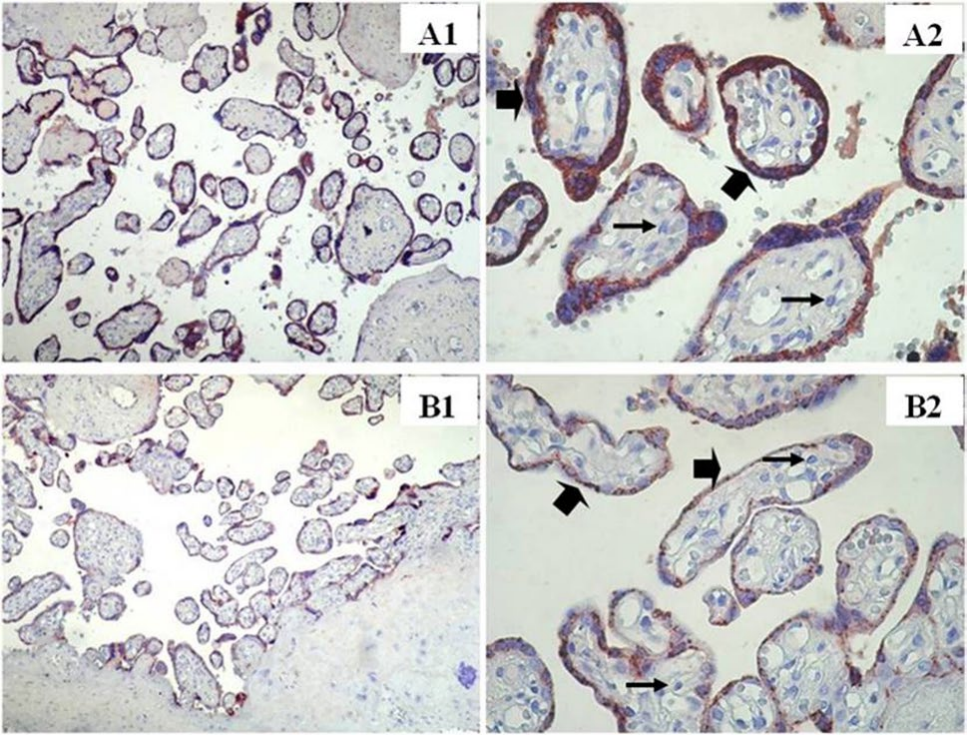
HPL immunostainings in the term placenta villi of Nonsmoker and smoker groups. Nonsmoker **A**, Smoker **B**. ➠:Villous syncytiotrophoblast cells, →: villous stromal cell. (A1,B1) X100, (A2,B2) X400

In the smoking group, weak IGF-1 immunoreactivity (Fig. 1B1, B2), moderate leptin immunoreactivity (Fig. 2B1, B2), and weak HPL immunoreactivity (Fig. 3B1, B2) were observed in both villous stromal cells and syncytiotrophoblasts.

In the non-smoking group, moderate to strong IGF-1 immunoreactivity was observed in villous stromal cells, whereas weak to moderate IGF-1 immunoreactivity was observed in syncytiotrophoblast cells (Fig. 1A1, A2). Leptin immunoreactivity was strong in villous stromal cells and moderate in syncytiotrophoblasts (Fig. 2A1, A2). HPL immunostaining was strong in syncytiotrophoblast cells but weak in villous stromal cells (Fig. 3A1, A2).

**Fig. 4:**
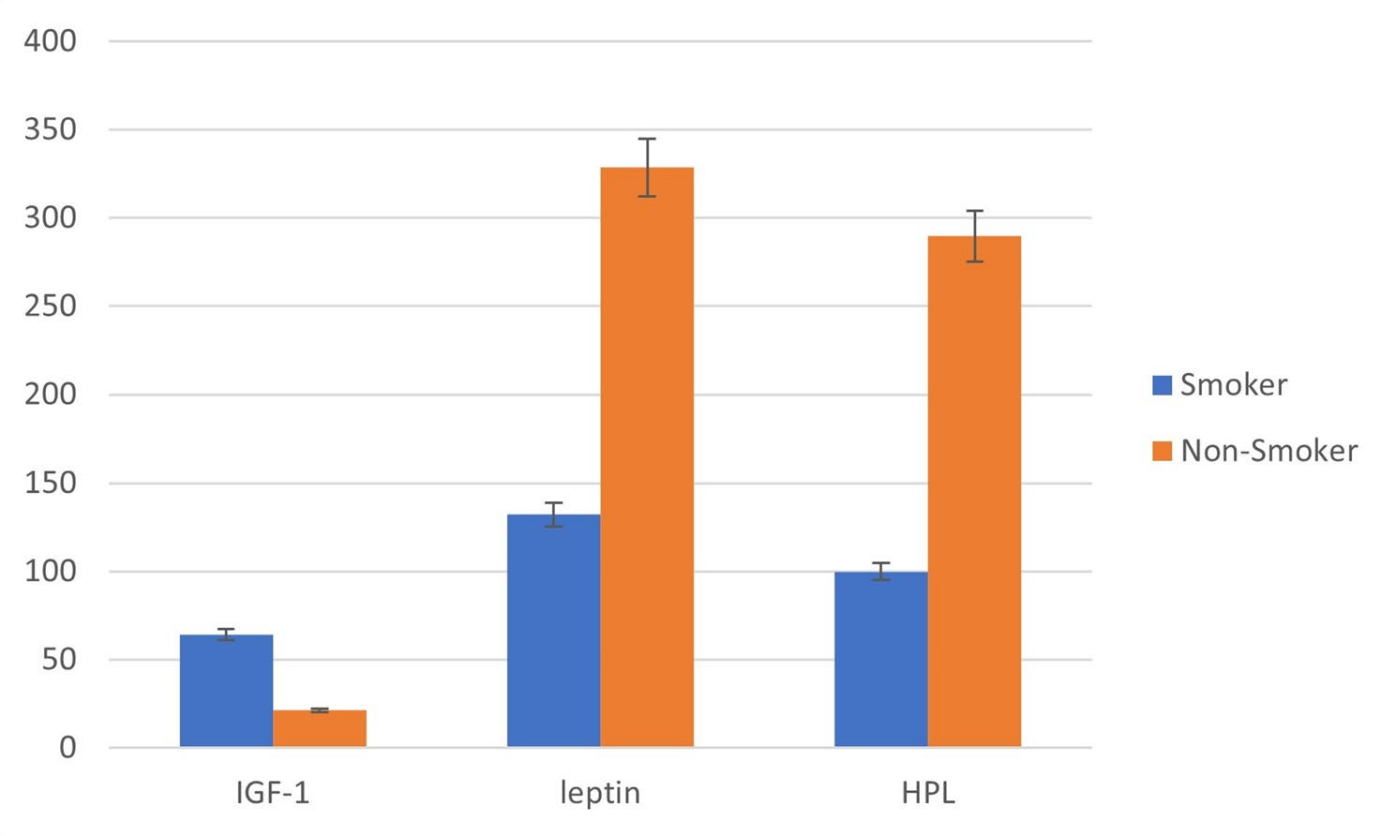
H-SCORE statistical analysis of IGF-1, leptin, HPL, immunoreactivity intensities for nonsmoker and smoker pregnant women. Values have been presented as mean ± SD

The staining intensity of IGF-1, leptin, and HPL was significantly higher in the non-smoking group than in the smoking group (*p* < 0.05) (Table 7; Fig. 4).

**Table 7:** Mean ± SD values of term placenta IGF-1, leptin, and HPL immunohistochemistry staining results between smoker and non-smoker pregnant mothers (*n* = 70)

Independent T- test HScore	Groups	*N*	Mean	Std. Deviation	t	df	*p*
IGF − 1	Smokers	35	64.1	21.87	−24.087	68	< 0.001**
	Nonsmokers	35	216.2	30.9			
Leptin	Smokers	35	132.2	24.2	21.663	68	< 0.001**
	Nonsmokers	35	328.5	47.7			
HPL	Smokers	35	99.9	19.5	−23.763	68	< 0.001**
	Nonsmokers	35	289.7	42.3			

Independent T test

(*) Indicates significant differences between groups

^**^p < 0.05

Comparison of placental immunohistochemical staining scores demonstrated significant differences in IGF-1, leptin, and human placental lactogen (HPL) expression between smoking and non-smoking pregnant mothers (Table 7).

Placental IGF-1 immunoreactivity was significantly lower in the smoking group compared with the non-smoking group (64.1 ± 21.87 vs. 216.2 ± 30.9, respectively; *t* = − 24.087, *df* = 68, *p* < 0.001).

Similarly, placental leptin immunohistochemical scores were significantly reduced in smokers compared with non-smokers (132.2 ± 24.2 vs. 328.5 ± 47.7, respectively; *t* = 21.663, *df* = 68, *p* < 0.001).

In addition, placental HPL immunoreactivity was markedly lower in the smoking group than in the non-smoking group (99.9 ± 19.5 vs. 289.7 ± 42.3, respectively; *t* = − 23.763, *df* = 68, *p* < 0.001).

### Umbilical cord blood biochemistry findings

A statistically significant difference was identified between the smoking status of pregnant mothers and their mean ± SD for leptin and HPL biochemistry findings. Umbilical cord blood analysis demonstrated significantly lower leptin and HPL levels in infants of smoking mothers than in those of non-smokers (*p* < 0.001 for both) (Table 8).

**Table 8:** Leptin and HPL biochemistry findings of smoker and nonsmoker pregnant mothers (*n* = 70)

Biochemistry Findings	Groups	*N*	Mean	Std. Deviation	t	df	*p*
Leptin (pg/ml)	Smokers	35	54.6	11.5	26.766	36.518	< 0.001**
	Nonsmokers	35	107.8	2.2			
HPL (pg/ml)	Smokers	35	0.9	0.6	−24.633	68	*< 0.001* **
	Nonsmokers	35	5.8	0.9			

Independent T test

(*) Indicates significant differences between groups

^**^p < 0.05

Umbilical cord blood biochemical analysis revealed significant differences in leptin and human placental lactogen (HPL) levels between infants born to smoking and non-smoking mothers (Table 8). Mean umbilical cord blood leptin levels were significantly lower in the smoking group compared with the non-smoking group (54.6 ± 11.5 pg/mL vs. 107.8 ± 2.2 pg/mL, respectively; *t* = 26.766, *df* = 68, *p* < 0.001).

Similarly, umbilical cord blood HPL levels were markedly reduced in the smoking group compared with the non-smoking group (0.9 ± 0.6 pg/mL vs. 5.8 ± 0.9 pg/mL, respectively; *t* = − 24.633, *df* = 68, *p* < 0.001).

These findings indicate that maternal smoking during pregnancy is associated with significantly lower leptin and HPL levels in umbilical cord blood.

## Discussion

In the present study, maternal smoking during pregnancy was found to be strongly associated with adverse fetal growth outcomes and altered placental endocrine function. Infants of mothers who smoked were considerably more likely to be classified as small for gestational age (SGA, ≤ 10th percentile) than those born to non-smoking mothers, underscoring the well-established link between prenatal smoking and impaired fetal growth. Importantly, by stratifying participants according to both smoking status and gestational age–adjusted birth weight percentiles, and by concurrently evaluating early breastfeeding practices, this study provided a more nuanced understanding of how smoking-related placental hormone dysregulation interacts with fetal growth status and postnatal feeding outcomes.

Birth weight is considered one of the primary indicators of fetal health and intrauterine development and is closely associated with placental hormone levels. Hormones produced by the placenta, such as insulin-like growth factor-1 (IGF-1), leptin, and human placental lactogen (HPL), have been shown in numerous studies to play key regulatory roles in fetal growth. Leptin contributes to fetal energy balance and growth, whereas IGF-1 supports tissue development by promoting cellular proliferation and differentiation. In contrast, HPL enhances fetal growth by modulating maternal metabolism to increase glucose availability to the fetus and by exerting prolactin-like effects. Alterations in the levels of these hormones have been consistently associated with fetal growth restriction [32]. Therefore, assessment of placental hormone expression is essential for understanding the biological mechanisms underlying impaired fetal growth.

By stratifying participants according to both maternal smoking status and gestational age–adjusted birth weight percentiles, the present study provides further insight into the interaction between prenatal smoking exposure and fetal growth in relation to placental endocrine function. The markedly reduced placental IGF-1 and HPL expression observed in the smoker–SGA group, together with the concomitant increase in placental leptin expression, suggests that maternal smoking may exacerbate placental endocrine dysfunction, particularly in pregnancies complicated by impaired fetal growth. The finding that lower placental IGF-1 expression is associated with SGA status is consistent with previous reports demonstrating reduced circulating IGF-I concentrations in neonates born to mothers who smoked during pregnancy and a positive association between IGF-I levels, birth weight, and neonatal length [33]. Given the central role of IGF-1 in placental nutrient transfer and fetal growth regulation, smoking-related suppression of IGF-1 may represent a key mechanistic pathway linking maternal smoking to fetal growth restriction. The observed alterations in placental leptin expression according to fetal growth status are also in agreement with existing evidence indicating that leptin is actively synthesized by trophoblast cells and exerts important endocrine and paracrine functions within the placenta. Increased placental leptin expression in growth-restricted pregnancies has been proposed to reflect a compensatory response to placental stress, hypoxia, or impaired nutrient transport, rather than an indicator of enhanced fetal growth potential [23].

In line with this, increased placental leptin transcript abundance has been reported in pregnancies complicated by intrauterine growth restriction (IUGR) and small for gestational age (SGA), supporting the association between leptin dysregulation and impaired fetal growth.

Similarly, human placental lactogen (HPL) is a key placental hormone whose secretion reflects placental mass and metabolic adaptation during gestation. Although the relationship between HPL and birth weight is complex, several studies have demonstrated significant associations between HPL levels, placental size, and gestational age–adjusted fetal growth, supporting the biological plausibility of HPL involvement in the regulation of fetal growth and nutrient supply [34, 35]. The reduced placental HPL expression observed in smoking mothers in the present study may therefore contribute to impaired fetal growth and adverse neonatal outcomes.

Most studies have focused on the protective effects of breast milk, and it is well established that these effects are modulated by environmental, cultural, and socioeconomic factors. The protective impact of breastfeeding is particularly pronounced in populations characterized by high infant mortality, poor sanitation, inadequate nutrition, and a high prevalence of tobacco exposure [36]. However, maternal tobacco smoking has been shown to adversely affect breast milk quality and composition, including alterations in taste, antioxidant capacity, long-chain polyunsaturated fatty acids, cytokine profiles, immunoglobulin content, and increased exposure to heavy metals [37]. Previous studies have demonstrated that exposure to tobacco smoke constituents through breast milk may negatively influence infant development and increase susceptibility to adverse health outcomes [38–40], including an association between heavy maternal smoking during pregnancy and lactation and an elevated risk of early-onset leukemia [41]. In addition, infants breastfed by smoking mothers have been reported to have a higher risk of colic [42], respiratory infections, and other health complications, potentially due to the transfer of nicotine and other toxic substances into colostrum and breast milk during critical periods of immune system maturation [43–49]. Smoking has also been associated with delayed lactogenesis II, which may interfere with early breastfeeding initiation and adequate colostrum intake [42]. Consistent with these observations, our study found that colostrum feeding rates were lower among smoking mothers (88.6%, *n* = 31) compared with non-smoking mothers (94.3%, *n* = 33), whereas formula supplementation was more frequent in the smoking group (11.4% vs. 5.7%). These findings suggest that maternal smoking may impair early lactational performance, potentially through reduced milk secretion and altered milk composition, thereby contributing to suboptimal breastfeeding practices during the early postnatal period and further compounding the risk of adverse outcomes in growth-restricted (SGA) infants.

When reviewing the literature on the timing of breastfeeding initiation, the World Health Organization (WHO) and UNICEF recommend initiating breastfeeding within the first hour after birth [50]. Early initiation of breastfeeding has been shown to play a critical role in reducing the risk of neonatal hypothermia during the immediate postnatal period [51], and breastfeeding support interventions have been demonstrated to positively influence the timing of initiation [52]. Recent studies, including a 2024 investigation in preterm infants, have further reported that early provision of mothers’ milk shortens the time to enteral feeding initiation and improves feeding-related outcomes [53, 54]. Consistent with these recommendations, our study demonstrated a marked delay in breastfeeding initiation among smoking mothers. Initiation of breastfeeding within the first hour postpartum was observed in only 57.1% (*n* = 20) of smokers, whereas 42.9% (*n* = 15) initiated breastfeeding during the second hour. In contrast, 94.3% (*n* = 33) of non-smoking mothers initiated breastfeeding within the first hour, and only 5.7% (*n* = 2) during the second hour. These findings indicate that maternal smoking during pregnancy is associated with delayed initiation of breastfeeding in the early postnatal period. Taken together, the delayed onset of breastfeeding observed in smoking mothers may be partly explained by smoking-related placental endocrine dysfunction, particularly the reduced expression of HPL and leptin, hormones that are known to influence maternal metabolic adaptation, lactogenesis, and the transition to effective milk secretion in the early postpartum period. Such endocrine disturbances may further compromise postnatal adaptation, especially in infants with impaired fetal growth (SGA), thereby linking prenatal smoking exposure, placental dysfunction, and suboptimal early feeding practices within a unified biological framework.

IGF-1 affects tissue growth and metabolism and plays a critical role in fetal growth and development [21, 55]. In a study conducted by Fleisch et al. in 2017, umbilical cord blood IGF-1 levels were compared among pregnant women who continued smoking during pregnancy, those with a history of smoking, and never-smokers. The lowest IGF-1 concentrations in cord blood were observed in women who continued to smoke throughout pregnancy [31]. Consistent with these findings, the present study demonstrated significantly reduced placental IGF-1 immunoreactivity in mothers who smoked during pregnancy.

Human placental lactogen (HPL) is exclusively produced during pregnancy, and its concentration in maternal serum increases in parallel with fetal and placental development. Although reduced HPL levels have been observed in women with higher body mass index (BMI), the extent to which prenatal health behaviors, including smoking, modulate HPL levels and influence infant birth weight remains incompletely understood [32]. Previous studies have reported an association between maternal smoking and reduced HPL levels. Carl et al. evaluated serial maternal serum HPL measurements during pregnancy and demonstrated that impaired fetal growth was associated with altered HPL trajectories, highlighting the relevance of HPL as a marker of placental and fetal development [56]. Similarly, Spira et al. reported that maternal smoking was associated with both reduced fetal weight and lower HPL levels [57]. Mochizuki et al. further demonstrated that heavy maternal smoking was linked to growth deficiency and decreased urinary estriol and HPL levels compared with non-smokers [58]. Consistent with these findings, the present study demonstrated significantly reduced placental HPL immunoreactivity and lower umbilical cord blood HPL levels in smoking mothers compared with non-smoking mothers, supporting the concept that prenatal tobacco exposure adversely affects placental endocrine function and compromises metabolic and hormonal support to the fetus, thereby contributing to impaired fetal growth and an increased risk of small-for-gestational-age (SGA) outcomes.

Leptin is a peptide hormone produced primarily by white adipose tissue and the placenta and plays a central role in the regulation of energy homeostasis, fetal growth, and metabolic adaptation during pregnancy [59–61]. Beyond its central effects on appetite and energy expenditure, leptin synthesized in the placenta exerts important endocrine and paracrine functions by supporting trophoblast proliferation, survival, and nutrient transport through the activation of intracellular signaling pathways such as JAK/STAT, MAPK, and PI3K [62–67]. Several studies have highlighted the association between placental leptin expression and fetal growth. Placental leptin has been proposed to reflect placental stress or adaptive responses in pregnancies complicated by impaired fetal growth, rather than directly promoting increased fetal size [68]. Pardo et al. reported that cord blood leptin levels were primarily related to gestational age and were not significantly altered by maternal smoking in term AGA newborns [68]. In contrast, Mantzoros et al. suggested that altered leptin concentrations in smoking pregnancies may be associated with reduced fetal growth [69]. In the present study, both placental leptin immunoreactivity and umbilical cord blood leptin levels were significantly lower in smoking mothers compared with non-smoking mothers. Importantly, when stratified according to gestational age–adjusted birth weight percentiles, leptin expression was closely associated with fetal growth category, supporting a link between smoking-related placental leptin dysregulation and small-for-gestational-age (SGA) status. The higher placental and cord blood leptin levels observed in non-smoking mothers are consistent with more favorable fetal growth patterns, whereas reduced leptin expression in smoking mothers likely reflects compromised placental endocrine adaptation in growth-restricted (SGA) pregnancies.

Taken together, these findings suggest that smoking-related alterations in placental leptin expression are more closely related to fetal growth status than to isolated changes in neonatal adiposity, and may represent a compensatory yet insufficient placental response to an adverse intrauterine environment.

One of the main limitations of this study is the lack of biochemical assessment of the quantity and composition of breast milk in smoking mothers. In addition, the relatively small sample size and the absence of direct measurements of nicotine and its metabolites in breast milk limit the interpretation and generalizability of the findings. As this study was conducted at a single center, future multicenter investigations with larger cohorts are warranted to improve external validity. Furthermore, although it is well established that female infants tend to have slightly lower birth weights than male infants, sex-specific differences in gestational age–adjusted fetal growth (SGA vs. non-SGA) were not specifically examined and should be addressed in future studies.

Another important limitation is the lack of pregnancy-specific tobacco use data in the 2022 statistics published by the Turkish Statistical Institute (TURKSTAT), despite strong evidence demonstrating the adverse effects of smoking during pregnancy and lactation. This gap underscores the need for more comprehensive, large-scale epidemiological surveillance of maternal smoking. Finally, experimental studies, including animal models and detailed prenatal histopathological and biochemical evaluation of placental and mammary tissues, may further elucidate the mechanistic pathways linking maternal smoking, placental endocrine dysfunction, impaired lactogenesis, and fetal growth restriction.

## Conclusion

Antenatal smoking is inversely associated with fetal growth and is known to exert detrimental effects on placental structure and endocrine function. In the present study, lower levels of IGF-1, leptin, and human placental lactogen (HPL) were observed in both placental tissue and umbilical cord blood of mothers who smoked during pregnancy compared with non-smoking mothers. In addition, placental immunostaining intensities of IGF-1, leptin, and HPL were significantly higher in the non-smoking group, indicating a more favorable placental endocrine profile.

With respect to early feeding outcomes, smoking mothers exhibited lower rates of breastfeeding initiation within the first hour after birth, reduced exclusive breastfeeding rates, and a higher prevalence of mixed feeding practices. Although direct causal pathways between placental hormone alterations and breastfeeding behavior were not specifically examined, the coexistence of smoking-related placental endocrine dysfunction and suboptimal early feeding patterns suggests that prenatal tobacco exposure may adversely affect both intrauterine adaptation and early postnatal metabolic and lactational processes.

Overall, these findings indicate that maternal smoking during pregnancy is associated with impaired placental endocrine function, an increased risk of small-for-gestational-age (SGA) status, and less favorable early breastfeeding practices. Together, these results underscore the critical importance of effective smoking cessation interventions during pregnancy, not only to improve fetal growth and placental function but also to promote optimal early postnatal nutrition and long-term infant health outcomes.

## Data Availability

The datasets used and/or analysed in the current study are available from the corresponding author upon reasonable request.

## References

[1] Simpson WJ. A preliminary report on cigarette smoking and the incidence of prematurity. Am J Obstet Gynecol. 1957;73(4):808–15. 13411046

[2] World Health Organization. WHO global report on trends in prevalence of tobacco use 2000–2030. Geneva: World Health Organization; 2024.

[3] Turkish Statistical Institute (TURKSTAT). Tobacco use statistics, 2022. Ankara: TUIK; 2022.

[4] Higgins S. Smoking in pregnancy. Curr Opin Obstet Gynecol. 2002;14(2):145–51. 11914691 10.1097/00001703-200204000-00007

[5] Slater L. Substance use in pregnancy. Pract Midwife. 2015;18(1):10–3. 26310086

[6] Salameh TN, Hall LA, Hall MT. Cigarette smoking cessation counselling in pregnant smokers with mental illness/substance use disorders. West J Nurs Res. 2023;45(3):234–41. 36196024 10.1177/01939459221127803 PMC9902998

[7] Zaganjor I, Kramer RD, Kofie JN, Sawdey MD, Cullen KA. Trends in smoking before, during, and after pregnancy in the united States from 2000 to 2020: pregnancy risk assessment monitoring system. J Womens Health. 2024;33(3):283–93. 10.1089/jwh.2023.0641 38153374

[8] Ruokolainen O, Ollila H, Laatikainen T, Pätsi SM, Carreras G, Gorini G, et al. Tobacco endgame measures and their adaptation in selected European countries: a narrative review synthesis. Tob Prev Cessat. 2024;10:1–12. 10.18332/tpc/186402 PMC11025294 38638446

[9] Shiverick K, Salafia C. Cigarette smoking and pregnancy I: ovarian, uterine and placental effects. Placenta. 1999;20(4):265–72. 10329346 10.1053/plac.1998.0377

[10] Andres RL, Day MC, editors. Perinatal complications associated with maternal tobacco use. Semin Neonatol. 2000;5:1–120. 10.1053/siny.2000.0025 10956448

[11] Macchi M, Bambini L, Franceschini S, Alexa ID, Agostoni C. The effect of tobacco smoking during pregnancy and breastfeeding on human milk composition: a systematic review. Eur J Clin Nutr. 2021;75(5):736–47. 33087893 10.1038/s41430-020-00784-3

[12] Branco J, Manuel AR, Completo S, Marques J, Rodrigues Antão R, Pinto Gago C, et al. Prevalence and predictive factors of exclusive breastfeeding in the first six months of life. Acta Med Port. 2023;36(6):416–23. 36947662 10.20344/amp.18692

[13] Salim YM, Stones W. Determinants of exclusive breastfeeding in infants of six months and below in Malawi: a cross-sectional study. BMC Pregnancy Childbirth. 2020;20(1):472. 32807130 10.1186/s12884-020-03160-y PMC7433092

[14] Otim ME, Omagino EK, Almarzouqi A, Rahman SA, Asante AD. Exclusive breastfeeding in the first six months: findings from a cross-sectional survey in Mulago Hospital, Uganda. Afr Health Sci. 2022;22(2):535–44. 36407345 10.4314/ahs.v22i2.62 PMC9652645

[15] Møll CA. Tobacco smoking: some hints of its biologic hazards. Ohio State Med J. 1950;46(12):1165–70. 14797254

[16] Thompson WB. Nicotine in breast milk. Am J Obstet Gynecol. 1933;26(5):662–7.

[17] Centers for Disease Control and Prevention (CDC). Tobacco use and breastfeeding: clinical guidance. Atlanta: CDC; 2023.

[18] Amir LH. Maternal smoking and reduced duration of breastfeeding: a review of possible mechanisms. Early Hum Dev. 2001;64(1):45–67. 11408108 10.1016/s0378-3782(01)00170-0

[19] Woodward A, Grgurinovich N, Ryan P. Breastfeeding and smoking hygiene: major influences on cotinine in urine of smokers’ infants. J Epidemiol Community Health. 1986;40(4):309–15. 3655623 10.1136/jech.40.4.309 PMC1052551

[20] Koutsaki M, Sifakis S, Zaravinos A, Koutroulakis D, Koukoura O, Spandidos DA. Decreased placental expression of hPGH, IGF-I and IGFBP-1 in pregnancies complicated by fetal growth restriction. Growth Horm IGF Res. 2011;21(1):31–6. 21212012 10.1016/j.ghir.2010.12.002

[21] Sferruzzi-Perri A, Owens J, Pringle K, Roberts C. The neglected role of insulin-like growth factors in the maternal circulation regulating fetal growth. J Physiol. 2011;589(1):7–20. 20921199 10.1113/jphysiol.2010.198622 PMC3021777

[22] Haschke F, van’t Hof MA, Euro-Growth Study Group. Euro-Growth references for breast-fed boys and girls: influence of breastfeeding and solids on growth until 36 months of age. J Pediatr Gastroenterol Nutr. 2000;31(Suppl 1):S60–71. 10896090 10.1097/00005176-200007001-00006

[23] Kochhar P, Manikandan C, Ravikumar G, Dwarkanath P, Sheela CN, George S, et al. Placental expression of leptin: fetal sex-independent relation with human placental growth. Eur J Clin Nutr. 2020;74(11):1603–12. 32382074 10.1038/s41430-020-0649-9

[24] RoghairRD, Colaizy TT, Steinbrekera B, Vass RA, Hsu E, Dagle D et al. Neonatal leptin levels predict early childhood developmental assessment scores of preterm infants. Nutrients. 2023;15(8). 10.3390/nu15081967. 10.3390/nu15081967 PMC10144252 37111184

[25] Ong KK, Preece MA, Emmett PM, Ahmed ML, Dunger DB. Size at birth and early childhood growth in relation to maternal smoking, parity and infant breastfeeding: a longitudinal birth cohort study. Pediatr Res. 2002;52(6):863–7. 12438662 10.1203/00006450-200212000-00009

[26] Sørensen S, von Tabouillot D, Schiøler V, Greisen G, Petersen S, Larsen T. Serial measurements of serum human placental lactogen and ultrasound evaluation of fetal growth. Early Hum Dev. 2000;60(1):25–34. 11054581 10.1016/s0378-3782(00)00101-8

[27] Spellacy WN, Buhi WC, Birk SA. The effect of smoking on serum human placental lactogen levels. Am J Obstet Gynecol. 1977;127(3):232–4. 835618 10.1016/0002-9378(77)90459-8

[28] Sarsmaz HY, Gürgen SG, Cansu A, Türkmen S, Gündüz A. The relationship between oxidative stress and apoptosis in ovarian histopathology induced by grayanotoxin-containing mad honey. Food Chem Toxicol. 2024;187:114634. 38582344 10.1016/j.fct.2024.114634

[29] Kharb S, Nanda S. Patterns of biomarkers in cord blood during pregnancy and preeclampsia. Curr Hypertens Rev. 2017;13(1):57–64. 28128050 10.2174/1573402113666170126101914

[30] Pardo IM, Geloneze B, Tambascia MA, Barros-Filho AA. Does maternal smoking influence leptin levels in term, appropriate-for-gestational-age newborns? J Matern Fetal Neonatal Med. 2004;15(6):408–10. 15280113 10.1080/14767050410001680046

[31] Fleisch AF, Rifas-Shiman SL, Rokoff LB, Hivert MF, Mantzoros CS, Oken E. Associations of maternal prenatal smoking with umbilical cord blood hormones: the project Viva cohort. Metabolism. 2017;72:18–26. 28641780 10.1016/j.metabol.2017.04.001 PMC5497769

[32] Garay SM, Sumption LA, John RM. Prenatal health behaviours as predictors of human placental lactogen levels. Front Endocrinol (Lausanne). 2022;13:946539. 36157466 10.3389/fendo.2022.946539 PMC9500170

[33] Vatten LJ, Nilsen ST, Odegård RA, Romundstad PR, Austgulen R. Insulin-like growth factor I and leptin in umbilical cord plasma and infant birth size at term. Pediatrics. 2002;109(6):1131–5. 12042554 10.1542/peds.109.6.1131

[34] Rassie K, Giri R, Joham AE, Teede H, Mousa A. Human placental lactogen in relation to maternal metabolic health and fetal outcomes: a systematic review and meta-analysis. Int J Mol Sci. 2022. 10.3390/ijms232415621. 36555258 10.3390/ijms232415621 PMC9779646

[35] Stern C, Schwarz S, Moser G, Cvitic S, Jantscher-Krenn E, Gauster M, et al. Placental endocrine activity: adaptation and disruption of maternal glucose metabolism in pregnancy and the influence of fetal sex. Int J Mol Sci. 2021;22(23):12722. 34884524 10.3390/ijms222312722 PMC8657775

[36] Zielinska MA, Hamulka J. Protective effect of breastfeeding on adverse health effects induced by air pollution: current evidence and possible mechanisms. Int J Environ Res Public Health. 2019;16:21. 10.3390/ijerph16214181 PMC6862650 31671856

[37] Napierala M, Mazela J, Merritt TA, Florek E. Tobacco smoking and breastfeeding: effects on the lactation process, breast milk composition and infant development. Environ Res. 2016;151:321–38. 27522570 10.1016/j.envres.2016.08.002

[38] Yilmaz G, Hizli S, Karacan C, Yurdakök K, Coşkun T, Dilmen U. Effect of passive smoking on growth and infection rates of breast-fed and non-breast-fed infants. Pediatr Int. 2009;51(3):352–8. 19400822 10.1111/j.1442-200X.2008.02757.x

[39] Dahlström A, Ebersjö C, Lundell B. Nicotine in breast milk influences heart rate variability in the infant. Acta Paediatr. 2008;97(8):1075–9. 18498428 10.1111/j.1651-2227.2008.00785.x

[40] Mennella JA, Yourshaw LM, Morgan LK. Breastfeeding and smoking: short-term effects on infant feeding and sleep. Pediatrics. 2007;120(3):497–502. 17766521 10.1542/peds.2007-0488 PMC2277470

[41] Ferreira JD, Couto AC, Pombo-de-Oliveira MS, Koifman S. Pregnancy, maternal tobacco smoking, and early age leukemia in Brazil. Front Oncol. 2012;2:151. 23162789 10.3389/fonc.2012.00151 PMC3494108

[42] Vlachou M, Kyrkou GA, Vivilaki V, Georgakopoulou VE, Katsaounou P, Kapetanaki A, et al. Tobacco smoke exposure and lactation. Cureus. 2024;16(11):e73651. 39677116 10.7759/cureus.73651 PMC11645517

[43] Mansouri B, Azadi NA, Sharafi K, Nakhaee S. Effects of active and passive smoking on selected trace element levels in human milk. Sci Rep. 2023;13(1):20756. 38007512 10.1038/s41598-023-48012-9 PMC10676413

[44] Faber T, Coffeng LE, Sheikh A, Reiss IK, Mackenbach JP, Been JV. Tobacco control policies and respiratory conditions among children presenting in primary care. NPJ Prim Care Respir Med. 2024;34(1):11. 38755181 10.1038/s41533-024-00369-8 PMC11099007

[45] Stepans MB, Wilkerson N. Physiologic effects of maternal smoking on breast-feeding infants. J Am Acad Nurse Pract. 1993;5(3):105–13. 8347401 10.1111/j.1745-7599.1993.tb00850.x

[46] Chulada PC, Arbes SJ Jr, Dunson D, Zeldin DC. Breast-feeding and the prevalence of asthma and wheeze in children: analyses from the Third National Health and Nutrition Examination Survey, 1988–1994. J Allergy Clin Immunol. 2003;111(2):328–36. 12589353 10.1067/mai.2003.127

[47] Halima BA, Sarra K, Kais R, Salwa E, Najoua G. Indicators of oxidative stress in weanling and pubertal rats following exposure to nicotine via milk. Hum Exp Toxicol. 2010;29(6):489–96. 19900975 10.1177/0960327109354440

[48] Klonoff-Cohen HS, Edelstein SL, Lefkowitz ES, Srinivasan IP, Kaegi D, Chang JC, et al. Passive smoking and tobacco exposure through breast milk and sudden infant death syndrome. JAMA. 1995;273(10):795–8. 7861574 10.1001/jama.1995.03520340051035

[49] Liebrechts-Akkerman G, Lao O, Liu F, van Sleuwen BE, Engelberts AC, L’Hoir MP, et al. Postnatal parental smoking: an important risk factor for SIDS. Eur J Pediatr. 2011;170(10):1281–91. 21404101 10.1007/s00431-011-1433-6 PMC3175033

[50] Arts M, Taqi I, Bégin F. Improving the early initiation of breastfeeding: the WHO–UNICEF breastfeeding advocacy initiative. BMJ. 2017;12(6):326–7. 10.1089/bfm.2017.0047 28509574

[51] Daniel E, Seif SA, Millanzi WC. Predictors of neonatal hypothermia within six hours of birth and preventive practices among postnatal mothers in Kilimanjaro region. PLoS One. 2024;19(11):e0313432. 39514561 10.1371/journal.pone.0313432 PMC11548749

[52] Patnode CD, Senger CA, Coppola EL, Iacocca MO. Interventions to support breastfeeding: updated evidence report and systematic review for the US preventive services task force. JAMA. 2025;333(17):1527–37. 40198081 10.1001/jama.2024.27267 PMC13521260

[53] Razzaghy J, Shukla VV, Gunawan E, Reeves A, Nguyen K, Salas AA. Early and exclusive enteral nutrition in very preterm infants. Arch Dis Child Fetal Neonatal Ed. 2024;109(4):378–83. 38135494 10.1136/archdischild-2023-325969 PMC11186726

[54] Umasekar U, Amboiram P, Balakrishnan U, Sirala Jagadeesh N. Effect of early establishment of full enteral feeding with exclusive mother’s own milk in preterm babies. BMJ Paediatr Open. 2025;9(1):eXXXX. 10.1136/bmjpo-2024-002931 PMC11749319 39773977

[55] Hiden U, Glitzner E, Hartmann M, Desoye G. Insulin and the IGF system in the human placenta of normal and diabetic pregnancies. J Anat. 2009;215(1):60–8. 19467150 10.1111/j.1469-7580.2008.01035.x PMC2714639

[56] Carl J, Christensen M, Mathiesen O. Human placental lactogen model for normal pregnancy. Placenta. 1991;12(3):289–98. 1754578 10.1016/0143-4004(91)90011-4

[57] Spira A, Philippe E, Spira N, Dreyfus J, Schwartz D. Smoking during pregnancy and placental pathology. Biomedicine. 1977;27(7):266–70. 588667

[58] Mochizuki M, Maruo T, Masuko K, Ohtsu T. Effects of smoking on the fetoplacental–maternal system during pregnancy. Am J Obstet Gynecol. 1984;149(4):413–20. 6203408 10.1016/0002-9378(84)90156-x

[59] Friedman JM. Leptin and the regulation of body weight. Keio J Med. 2011;60(1):1–9. 21460597 10.2302/kjm.60.1

[60] Myers MG Jr, Cowley MA, Münzberg H. Mechanisms of leptin action and leptin resistance. Annu Rev Physiol. 2008;70:537–56. 17937601 10.1146/annurev.physiol.70.113006.100707

[61] Pelleymounter MA, Cullen MJ, Baker MB, Hecht R, Winters D, Boone T, et al. Effects of the obese gene product on body weight regulation in ob/ob mice. Science. 1995;269(5223):540–3. 7624776 10.1126/science.7624776

[62] Morton GJ, Cummings DE, Baskin DG, Barsh GS, Schwartz MW. Central nervous system control of food intake and body weight. Nature. 2006;443(7109):289–95. 16988703 10.1038/nature05026

[63] Park HK, Ahima RS. Physiology of leptin: energy homeostasis, neuroendocrine function and metabolism. Metabolism. 2015;64(1):24–34. 25199978 10.1016/j.metabol.2014.08.004 PMC4267898

[64] Labbé SM, Caron A, Lanfray D, Monge-Rofarello B, Bartness TJ, Richard D. Hypothalamic control of brown adipose tissue thermogenesis. Front Syst Neurosci. 2015;9:150. 26578907 10.3389/fnsys.2015.00150 PMC4630288

[65] Sagawa N, Yura S, Itoh H, Kakui K, Takemura M, Nuamah MA, et al. Possible role of placental leptin in pregnancy. Endocrine. 2002;19(1):65–71. 12583603 10.1385/ENDO:19:1:65

[66] Rumer KK, Sehgal S, Kramer A, Bogart KP, Winn VD. Effects of leptin on human cytotrophoblast invasion. Front Endocrinol (Lausanne). 2024;15:1386309. 38846494 10.3389/fendo.2024.1386309 PMC11154010

[67] Maymó JL, Pérez-Pérez A, Gambino Y, Calvo JC, Sánchez-Margalet V, Varone CL. Leptin gene expression in the placenta: regulation in trophoblast proliferation and survival. Placenta. 2011;32(Suppl 2):S146–53. 21303721 10.1016/j.placenta.2011.01.004

[68] Pardo I, Geloneze B, Tambascia M, Barros-Filho A. Maternal smoking and leptin levels in term newborns. J Matern Fetal Neonatal Med. 2004;15(6):408–10. 15280113 10.1080/14767050410001680046

[69] Mantzoros CS, Varvarigou A, Kaklamani VG, Beratis NG, Flier JS. Effect of birth weight and maternal smoking on cord blood leptin concentrations. J Clin Endocrinol Metab. 1997;82(9):2856–61. 9284710 10.1210/jcem.82.9.4248

